# Top-100 Most Cited Publications Concerning Network Pharmacology: A Bibliometric Analysis

**DOI:** 10.1155/2019/1704816

**Published:** 2019-08-01

**Authors:** Cuncun Lu, Zhitong Bing, Zhijiang Bi, Ming Liu, Tingting Lu, Yangqin Xun, Zhipeng Wei, Kehu Yang

**Affiliations:** ^1^Evidence-Based Medicine Center, School of Basic Medical Sciences, Lanzhou University, Lanzhou, China; ^2^Evidence-based Social Science Center, School of Public Health, Lanzhou University, Lanzhou, China; ^3^Gansu University of Traditional Chinese Medicine, Lanzhou, China; ^4^Editorial Department of Library and Information, Gansu Province Library, Lanzhou, China

## Abstract

**Background:**

Network pharmacology (NP) has become an increasingly important focus in the drug research field over the past decade. However, no study to date has mapped the current status of NP. Therefore, we performed a bibliometric study to evaluate the top 100 cited papers on NP.

**Methods:**

We searched the Web of Science Core Collection from its inception to February 25, 2019, using the terms “network pharmacology” and “systems pharmacology.” Key data, including title, publication year, number of citations, authors, countries/regions, organizations, and journals, were retrieved and analyzed using Excel 2016 and VOSviewer 1.6.10.

**Results:**

The total number of citations for the 100 cited papers ranged from 21 to 1,238, published in 53 journals, from 2005 to 2017. The top three journals with the most publications on NP were *Clinical Pharmacology & Therapeutics* (*n* = 8, IF2017 = 6.544), *Journal of Ethnopharmacology* (*n* = 8, IF2017 = 3.115), and *PLoS One* (*n* = 7, IF2017 = 2.766). Most published articles were from the USA (*n* = 41) and China (*n* = 35). The most active author was *Wang Yonghua* from the Northwest A&F University, and of the 100 publications, 14 listing his name. The most frequently used substantive terms included “drug discovery,” “traditional Chinese medicine (TCM),” “in-vitro,” “cancer,” and “cardiovascular disease.” *Conclusions*. The USA and China made the greatest contribution to NP research. The current NP research mainly focused on NP methods (including experimental validation) and using them to explore the molecular mechanisms of TCM for some critical diseases such as cardiovascular diseases and cancers. Furthermore, we believe some guidelines should be developed to regulate NP studies.

## 1. Introduction

Network pharmacology (NP, also well known as systems pharmacology) is a novel method for drug discovery, combining systems biology, omics, computational biology, and bioinformatics to explore the potential mechanisms of drug activity through conetwork analyses [1–3]. NP can help to understand the polypharmacology of a drug and its effect on biological networks to improve drug efficacy [4]. In traditional Chinese medicine (TCM), herbs used for disease treatment are also considered to have multiple targets and pathways [5, 6]. The advent of NP has afforded new methods to identify particular targets of the active complex ingredients of herbs used in TCM and their interactions in the context of molecular networks [1, 3, 5]. To date, two common options for conducting NP are used. The first method, classic NP, involves the downloading of hundreds of herbal compounds from a database, or combining literature, and the mapping of a body of targets [6]. The second method involves the integration of experimental data to validate the computational results [7, 8]. Recently, many studies on NP for TCM have been published in various journals [6–11]. For example, Gao et al. [8] employed the NP and experimental methods, and they found *MMP2*, *CASP3*, *MYC,* and *REG1A* to be important targets in the Kushen injection for hepatocellular carcinoma.

**Figure 1:**
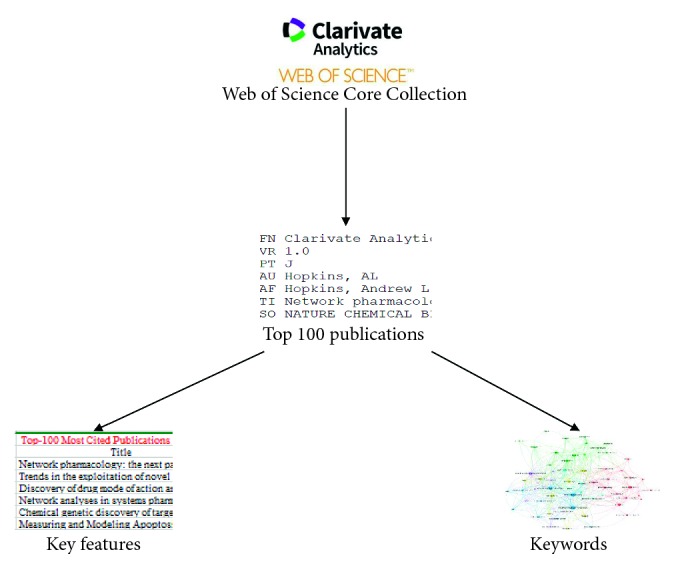
Workflow of the bibliometric study.

Bibliometrics studies use a set of methods to quantify published scientific knowledge [12, 13] and can be used to summarize the status quo and development trends of a specific disease or research field, providing ideas and directions for future research [14, 15]. Bibliometric approaches are commonly used in many research fields to map their foci and trends [16–18]. The Web of Science Core Collection (WoSCC) is the most commonly used platform for bibliometric analyses [19]. To date, there has been no comprehensive review of studies on NP using bibliometric methods. Therefore, herein, we designed a bibliometric study to analyze the 100 most cited papers on NP using data from WoSCC. The data provide a holistic perspective on the research status of NP by analyzing the publication years, number of citations, authors, countries/regions, organizations, journals, and other key features of these papers. The workflow of the bibliometric study on NP research is shown in Figure 1.

## 2. Methods

### 2.1. Data Sources

The 100 most cited NP-related papers were retrieved from the WoSCC database, and the time span was set from its inception to February 25, 2019, at Lanzhou University, Lanzhou, Gansu, China. The data were obtained on the same day to avoid bias. The search strategy was as follows: TS = (“network pharmacology” OR “systems pharmacology”) with only publications indexed as “ARTICLE” or “REVIEW” being included. All hits were ranked by the number of citations to identify the top 100 cited papers.

### 2.2. Data Extraction

Two independent authors assessed each identified paper to ensure that the study referred to NP, and a third author corroborated the results. Information was collected by combining the reports from the WoSCC database and manual supplementation, including title, accession number, publication year, publication types, number of citations, authors, countries/regions, organizations, journals, and their impact factors (IFs).

### 2.3. Statistical Analysis

Microsoft Excel 2016 software was used for descriptive statistical analyses of the publication year, publication types, number of citations, authors, countries/regions, organizations, and journals and their IFs (from 2017 Journal Citation Reports of Clarivate Analytics). VOSviewer 1.6.10 [20] software was used to map the network of keyword co-occurrence (unit of analysis was set as “All keywords”). In the terms' map, each bubble represented a term, the size of the bubble indicated how often it appeared. The color of a bubble indicated the special one cluster. If they appeared together in any of the 100 papers, a line connected the two bubbles; the thicker the line, the greater the number of times they appeared together. Furthermore, the greater the frequency of two terms appearing together, the closer the two bubbles would be. The terms map appears in at least three times of the 100 papers.

## 3. Results

The 100 most cited papers related to NP identified from the WoSCC included 62 original articles and 38 review articles. The total number of citations of a paper ranged from 21 to 1,238, with the average number of citations being 77.06 (7,706/100). Nearly one-fifth of articles (*n* = 18) had more than 100 citations and only one paper, published in *Nature Chemical Biology* (IF = 13.843) and authored by Andrew L Hopkins from the University of Dundee in 2008, was cited more than 1,000 times [1]. Supplementary Table S1 presents the data for the top 100 cited papers assessed in the present study.

### 3.1. Year of Publications

**Figure 2:**
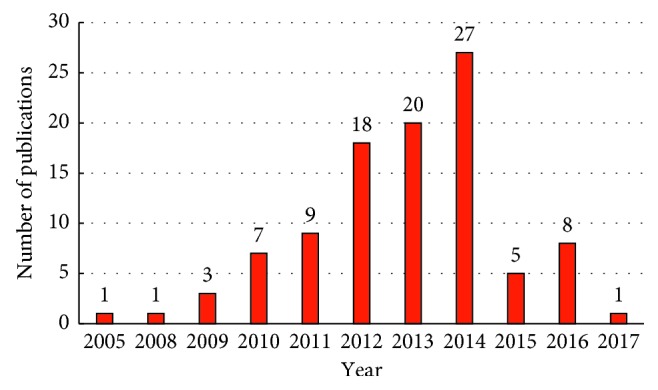
Year of the 100 most cited papers.

The papers included in the present study were all published between 2005 and 2017, but no studies from 2006 to 2007 were included. Therefore, the average number of years since 2005 was approximately 9. Furthermore, more than 10 published studies were found only in 2012, 2013, and 2014, with the greatest number of papers being published in 2014.  Figure 2 presents the distribution of the top 100 cited papers related to NP.

### 3.2. Distribution of Countries/Regions

The 100 most cited papers related to NP research were contributed by 25 countries/regions (Table S1). We found 14 countries/regions contributed to at least 2 NP publications each (Table 1); the greatest number of publications originated from the USA (*n* = 41), followed by China (*n* = 35) and England (*n* = 11). Finland, Germany, and the Netherlands contributed the same number of papers (*n* = 4).

### 3.3. Distribution of Organizations

More than 100 organizations (Table S1) made contributions to NP research, with 17 organizations contributing to at least 3 NP publications each (Table 2). Northwest A&F University, China, published 14% of the top 100 studies and ranked first, followed by Icahn School of Medicine at Mount Sinai, USA (10%, 10/100), Chinese Academy of Sciences, China (9%, 9/100), Dalian University of Technology, China (7%, 7/100), China Academy of Chinese Medical Sciences, China (5%, 5/100), and Harvard University, USA (5%, 5/100).

### 3.4. Distribution of Authors

The list of authors that published at least 5 NP publications each is presented in Table 3, with a total of 11 such authors. At the leading position in this list was Wang Yonghua, from the Northwest A&F University, who published 14 of the top 100 papers, followed by Yang Ling from the Chinese Academy of Sciences, who published 9 papers. Meanwhile, 7 other authors of the 11 with 5 or 6 NP studies were from the same university, Northwest A&F University. The other two authors Li Yan and Iyengar Ravi were from Dalian University of Technology and Icahn School of Medicine at Mount Sinai, respectively, with 7 NP studies each.

### 3.5. Distribution of Journals

Overall, the top 100 cited papers on NP were published in 53 academic journals (Table S1).  Table 4 summarizes the journals (*n* = 20) where at least two studies concerning NP were published. The IF range for these journals was from 2.064 (*Evidence-Based Complementary and Alternative Medicine*) to 50.167 (*Nature Reviews Drug Discovery*). Most of these are published in the USA (55%, 11/20) and England (35%, 7/20). *Clinical Pharmacology & Therapeutics* (IF2017 = 6.544) and *Journal of Ethnopharmacology* (IF2017 = 3.115) published the greatest number of papers (8%, 8/100), followed by *PLoS One* (7%, 7/100; IF2017 = 2.766) and *Molecular Biosystems* (5%, 5/100; IF2017 = 2.759).

### 3.6. Co-Occurrence of Keywords

**Figure 3:**
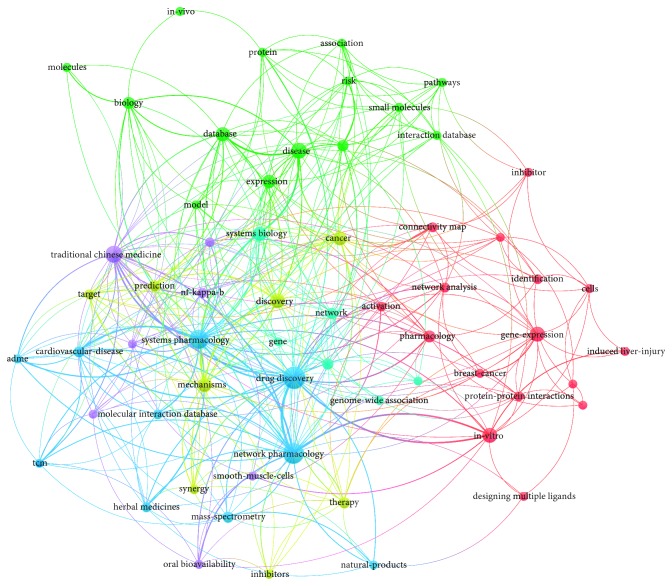
Network visualization of keywords of the 100 most cited papers.

In all, 61 terms appeared 3 times or more in the titles or abstracts of the top 100 papers related to NP (Figure 3). For example, “drug discovery” appeared 21 times; “traditional Chinese medicine” appeared 12 times, “in-vitro” appeared 9 times, “cancer” appeared 8 times, and “cardiovascular disease” appeared 5 times. Accordingly, the terms or phrases associated with NP are divided into six clusters, represented by six colors (azure, yellow, purple, green, light blue, and red). From the results of co-occurrences, current NP research was shown to be mainly focused on drug discovery, TCM, cardiovascular diseases, cancers, pathways, network analysis, gene expression, protein-protein interactions, oral bioavailability, and *in vitro* experiments, with these topics being regarded as the current research hotspots in the field of NP. The terms that appeared at least five times are listed in Table 5.

## 4. Discussion

Herein, we identified and analyzed the 100 most cited papers related to NP research published from 2005 to 2017. This bibliometric study aimed to evaluate the research status and trends in the field of NP research.

The USA, China, and England published more than 10 NP publications each, whereas a further 11 countries published only 2–4 NP studies each between 2005 and 2017. It is likely that the more common use of TCM in China than in other countries led to the larger number of studies published along with the fact that the USA and England have more advanced biotechnologies and broader cooperation. Northwest A&F University (China) contributed the most papers, likely due to its establishment of a TCM database, TCMSP [5], and the university may have a more profound research foundation. Furthermore, Professor Wang Yonghua from Northwest A&F University published the greatest number of papers and ranked the first in the all authors of top 100 cited papers.

The total number of citations for the top 100 cited papers ranged from 21 to 1,238, and only one paper was cited more than 1,000 times, considering that NP was the next paradigm in drug discovery [1]. Compared with the number of citations in other specialties [15, 21], this number of citations is very low, likely due to the relative novelty of the research topic. However, considering the importance of NP for the development of TCM and drug discovery, increasing the funding and number of researchers involved is of great importance for the development of NP. The top 100 cited papers were published in 53 journals, among which four journals (*Clinical Pharmacology & Therapeutics*, *Journal of Ethnopharmacology*, *PLoS One*, and *Molecular Biosystems*) published a total of 28 papers, almost one-third of the total number of papers. Furthermore, another large journal group of 16 journals published 39 of the 100 papers; the remaining papers were published by 33 other journals. Most of these journals are issued by the USA and England and are generally from the field of pharmacology or bioinformatics, on which NP research and current applications are focused.

According to the results of keyword co-occurrence, the 100 most cited papers covered the main topics of NP research, including its applications and common research methods. Drug discovery, TCM, cardiovascular diseases, cancers, pathways, network analysis, gene expression, protein-protein interactions, oral bioavailability, and *in vitro* experiments, being regarded as the current research hotspots. Similarly, Yeung et al. [22] analyzed 100 most cited papers in ethnopharmacology and highlighted the importance of traditional medicine in medicine and food science. Pathway and network analysis and protein-protein interactions are common methods for NP [6–8, 23]. Oral bioavailability [24] is an essential parameter for drug absorption and utilization; therefore, NP studies commonly use this alone or in combination with other parameters, including drug likeness, Caco-2 permeability, and drug half-life, as filters to select compounds from herbs [6, 7, 25]. Other methods, such as molecular docking, are also used in many NP studies [6, 23]. Tao et al. [26] used NP methods finding that *Radix Curcumae* and *Fructus Gardeniae* shared the common targets, demonstrated the synergistic effects of *Radix Curcumae* formula for cardiovascular diseases, and explained the prescription rule (*Jun-Chen-Zuo-Shi*) of TCM for diseases. Moreover, Lyu et al. [9] employed NP and experiment methods that found antiendothelial inflammation was the common mechanism of Danhong injection for stroke and coronary artery disease. Bing et al. [6] used NP methods to indicate that *licochalcone a* and *beta-sitosterol* in Fuzhengkangai were active compounds to overcome drug resistance. Gong et al. [25] found that *TTR* might be a potential treating target of hepatocellular carcinoma by using NP methods. Therefore, we can conclude that current NP research mainly focused on NP methods (including experimental validation) and using them to explore the molecular mechanisms of TCM for some critical diseases such as cardiovascular diseases and cancers. However, we found various existing designs and reports on NP, with some concerns regarding the development of NP due to the lack of guidelines on the design, conduct, interpretation, and reporting of NP studies. Additionally, similar to the early period of clinical medicine research, there is a lack of evidence-based medicine and its useful measures [27–29], with a vast amount of inconsistent evidence leading to confusion for clinicians and researchers. Therefore, we suggest that corresponding methodological and reporting guidelines should be developed to regulate the design and reporting of NP studies.

Our study has some advantages. First, to the best of our knowledge, this is the first study using the bibliometric method to summarize the status and development of the top 100 cited papers related to NP. Second, the top 100 cited papers related to NP analyzed in this study could be considered as indispensable for NP development. Third, the key data were all retrieved by combining the reports from WoSCC database and manual supplementation, thus ensuring the accuracy and completeness of the data. However, it had limitations similar to those of other bibliometric studies, namely, the limited search involving only the WoSCC database, with other common medicine databases, such as PubMed, Embase, and Scopus, not searched. Thus, some influential papers may have been missed. However, we note that the WoSCC database is the most used database for bibliometric analysis [19]. Second, it is logical that the citation frequency of studies published earlier should be greater than that of recent studies, therefore introducing bias. Third, the citation frequency of papers is influenced by factors such as the journals' IF, authors, and organizations; indeed, the academic influence of studies cannot be reflected only by the number of times they are cited.

## 5. Conclusion

A bibliometric study was performed for the top 100 cited papers related to NP. These papers were published from 2005 to 2017 in various journals. Based on results from bibliometric software and our prior knowledge, we found that the current NP research mainly focused on NP methods (including experimental validation) and using them to explore the molecular mechanisms of TCM for some critical diseases such as cardiovascular diseases and cancers. The USA and China published the most highly cited papers on NP. Professor *Wang Yonghua* from Northwest A&F University was the most published author. Generally, this bibliometric study provides, for the first time, insights into the historical developments and research status of NP studies, and we suggest that corresponding methodological and reporting guidelines should be developed to regulate the development of NP studies.

## Figures and Tables

**Table 1:** Countries/regions of the 100 most cited papers (record ≥ 2).

Country/region	Record
USA	41
China	35
England	11
Finland	4
Germany	4
Netherlands	4
Sweden	3
Australia	2
Belgium	2
Denmark	2
France	2
Italy	2
Scotland	2
Spain	2

**Table 2:** Organizations of the 100 most cited papers (record ≥ 3).

Organization	Record
Northwest A&F University	14
Icahn School of Medicine at Mount Sinai	10
Chinese Academy of Sciences	9
Dalian University of Technology	7
China Academy of Chinese Medical Sciences	5
Harvard University	5
University of Helsinki	4
Tsinghua University	4
University of Manchester	4
Peking University	4
Beijing University of Chinese Medicine	4
US-Food and Drug Administration	3
University of Pittsburgh	3
University of California San Diego	3
University at Buffalo	3
Northwestern University	3
Hong Kong Baptist University	3

**Table 3:** Authors of the 100 most cited papers (record ≥ 5).

Author	Organization	Record
Wang Yonghua	Northwest A&F University	14
Yang Ling	Chinese Academy of Sciences	9
Li Yan	Dalian University of Technology	7
Iyengar Ravi	Icahn School of Medicine at Mount Sinai	7
Tao Weiyang	Northwest A&F University	6
Xu Xue	Northwest A&F University	6
Zhou Wei	Northwest A&F University	6
Wang Xia	Northwest A&F University	5
Huang Chao	Northwest A&F University	5
Wang Jinan	Northwest A&F University	5
Li Bohui	Northwest A&F University	5

**Table 4:** Journals of the 100 most cited papers (record ≥ 2).

Journal	Record	Country/region	Impact factor (2017)
Clinical Pharmacology & Therapeutics	8	USA	6.544
Journal of Ethnopharmacology	8	Ireland	3.115
PLoS One	7	USA	2.766
Molecular Biosystems	5	England	2.759
Evidence-Based Complementary and Alternative Medicine	4	USA	2.064
Nucleic Acids Research	4	England	11.561
Annual Review of Pharmacology and Toxicology	3	USA	13.295
Current Pharmaceutical Design	3	United Arab Emirates	2.757
Drug Discovery Today	3	England	6.848
Analytical Chemistry	2	USA	6.042
Bioinformatics	2	England	5.481
Briefings in Bioinformatics	2	England	6.302
Cancer Research	2	USA	9.13
Chemistry & Biology	2	USA	5.915
Expert Opinion on Investigational Drugs	2	England	3.883
Nature Chemical Biology	2	USA	13.843
Nature Reviews Drug Discovery	2	England	50.167
PLoS Computational Biology	2	USA	3.955
Science Translational Medicine	2	USA	16.71
Wiley Interdisciplinary Reviews-Systems Biology and Medicine	2	USA	3.709

**Table 5:** The keywords in the titles or abstracts of the 100 most cited papers (occurrence ≥ 5).

Keyword	Occurrence	Link strength
Drug discovery	21	74
Network pharmacology	18	69
Systems pharmacology	15	64
Traditional Chinese medicine	12	46
Disease	10	28
Discovery	9	26
Gene-expression	9	18
In-vitro	9	23
Cancer	8	33
Database	8	29
Mechanisms	8	29
Systems biology	8	28
Expression	7	17
Biology	6	18
Pharmacology	6	16
Cardiovascular-disease	5	28
Mass-spectrometry	5	15
Myocardial-infarction	5	21
Network	5	17
Prediction	5	21
Signaling pathways	5	16
Therapy	5	16

## Supplementary Materials

Supplementary Materials Supplementary Table S1: the main data of the top 100 cited papers assessed in the present study.
